# Supplementary material for: Integrative Analysis of the Nasal Microbiota and Serum Metabolites in Bovines with Respiratory Disease by 16S rRNA Sequencing and Gas Chromatography/Mass Selective Detector-Based Metabolomics
Source: Int J Mol Sci. 2022 Oct 10;23(19):12028. doi: 10.3390/ijms231912028 (PMC9569885; doi:10.3390/ijms231912028)
Supplement: Supplementary file 1 [file ijms-23-12028-s001.zip › Supplementary Figures.pdf]

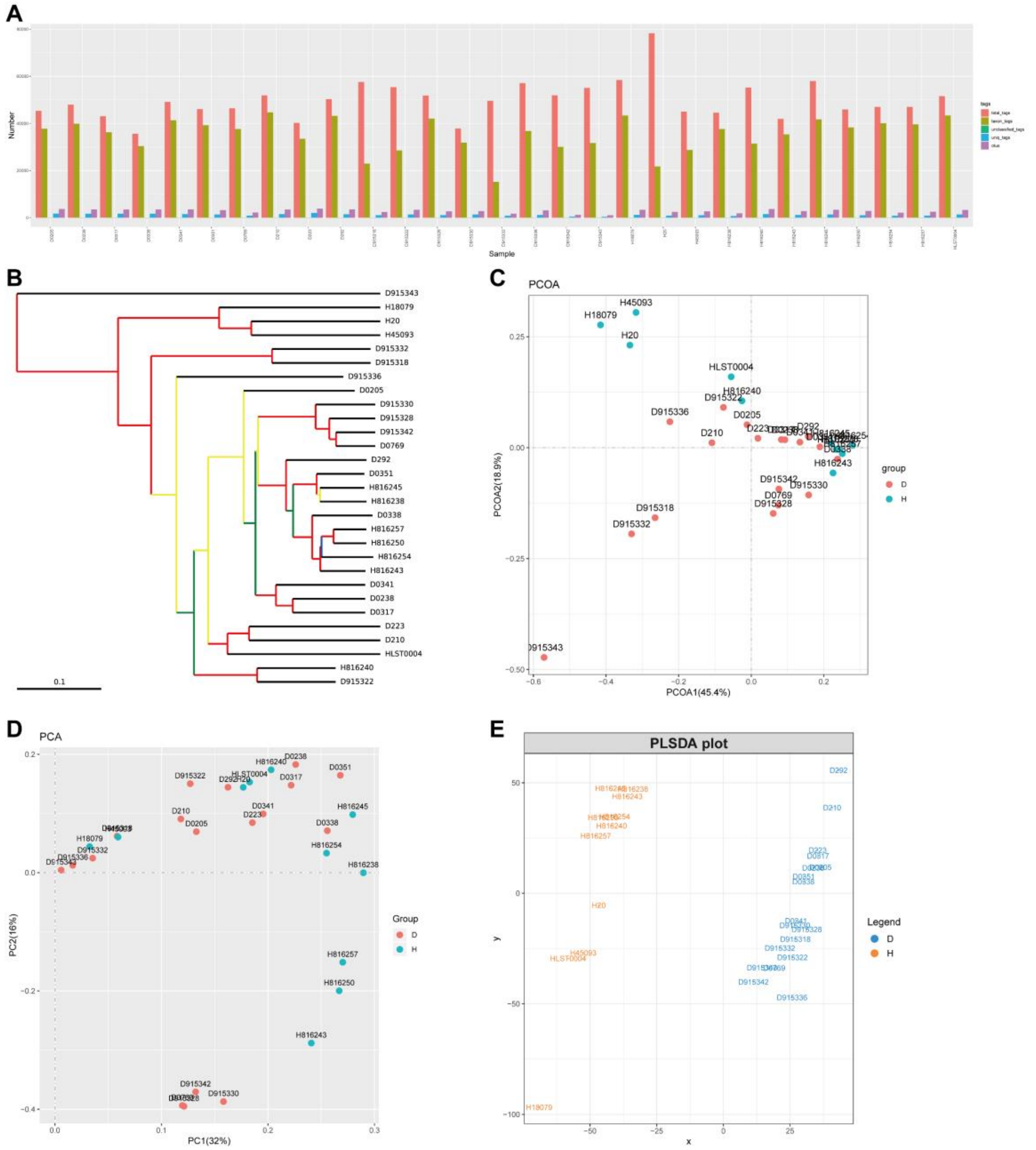

Figure S1. Diversity analysis between healthy cattle and cattle with respiratory disease. (A) The OUT clusters and annotation of each sample. (B) The evolution tree displays the similarity of whole samples. (C) Principal coordinate analysis (PCoA) using the weighted-UniFrad algorithm of beta diversity. (D) PCoA using Weighted-UniFrad of beta diversity. (E) PLS-DA analysis on the OUTs classification information of each sample. OUT, operational taxonomic unit.

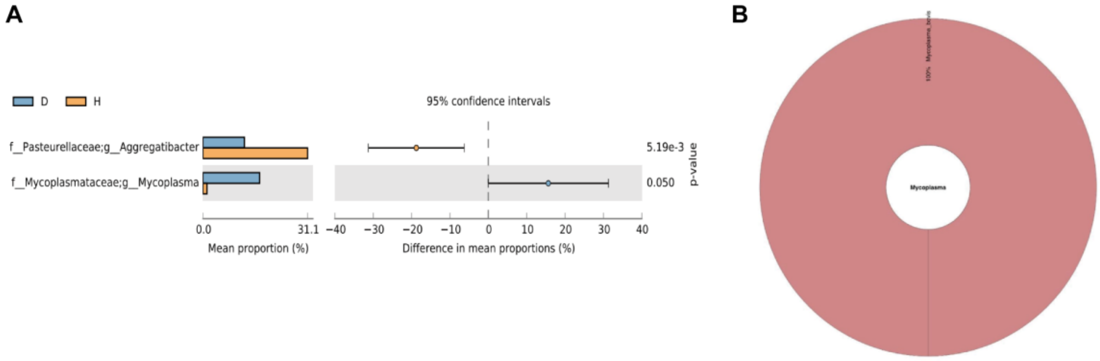

Figure S2. Identification of key pathogenic bacteria. (A) Student's t-test analysis of key bacterial types with significant differences between diseased D and healthy H groups. (B) Species annotation of the whole samples by Metagenomic sequencing.

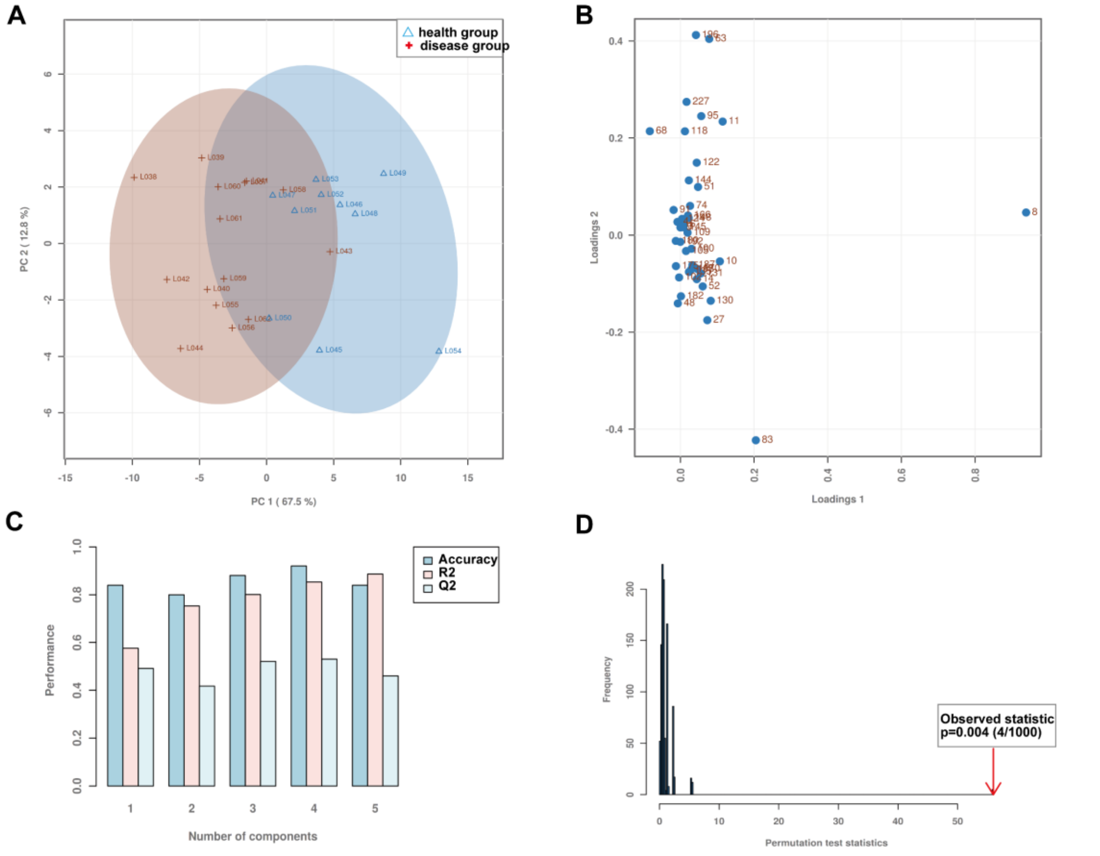

Figure S3. Unsupervised principal component analysis (PCA). Scores plot (A) and Loading plot (B) of GC-MSD profiled plasma from healthy and BRD cattle. (C) Cross-validation diagram of PLS-DA model. In this model, R2: 0.8535, Q2: 0.5300. (D) Permutation test compared with PLS-DA model. GC-MSD, gas chromatography/mass selective detector; BRD, bovine respiratory disease; PLS-DA, partial least-squares discriminant analysis.

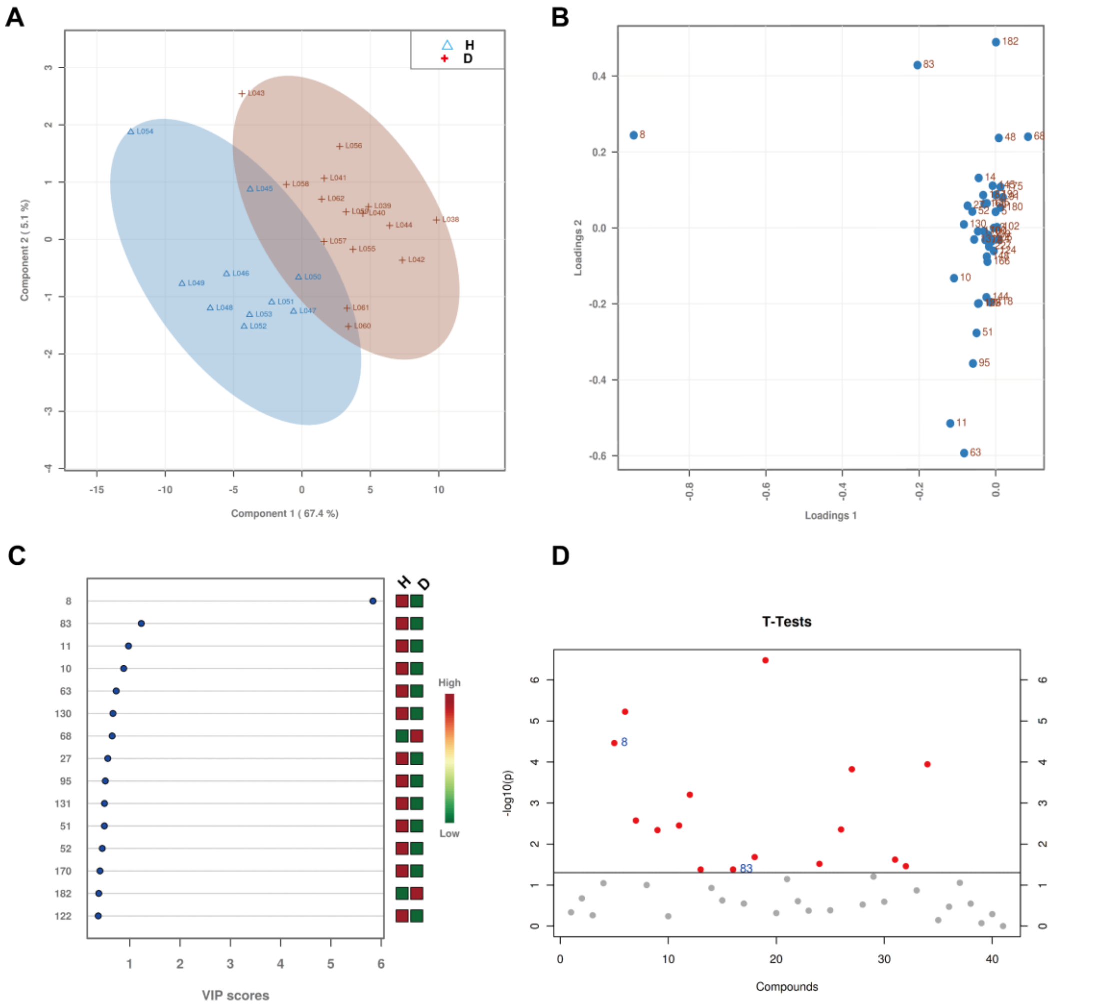

Figure S4. Evaluation of metabolites in bovine serum. Supervised PLS-DA scores plot (A) and loading plot (B) of GC-MSD profiled plasma from healthy and BRD cattle. (C) VIP of PLS-DA for different metabolites. Larger VIP scores are associated with a greater contribution to distinguishing the samples. The criteria for significance included VIP scores >1. (D) Statistical analysis (Student's t-test). Each dot represents a metabolite, and the solid line represents the threshold line of P = 0.05. Grey dots represent metabolites with P > 0.05, red dots represent metabolites with P < 0.05. The dots mark the VIP >1 metabolites obtained by PLS-DA analysis. PLS-DA, partial least-squares discriminant analysis; GC-MSD, gas chromatography/mass selective detector; BRD, bovine respiratory disease; VIP, variable importance in projection. D: bovines with respiratory disease, H: healthy bovines.
